# Safety, Tolerability, and Pharmacokinetics of Galcanezumab, an Anti‐CGRP Antibody, in Healthy Chinese Participants

**DOI:** 10.1002/cpdd.1599

**Published:** 2025-09-12

**Authors:** Jingjing Wang, Nanyang Li, Jinjie He, Jing Zhang, Lili Wang, William Kielbasa, Chenxi Qian, Yanjie Zhang, Liang Wang

**Affiliations:** ^1^ Clinical Pharmacology Research Center Huashan Hospital Fudan University Shanghai China; ^2^ Eli Lilly and Company Indianapolis IN USA; ^3^ Eli Lilly and Company Shanghai China; ^4^ Deparment of Neurology Huashan Hospital Fudan University Shanghai China

**Keywords:** Chinese, galcanezumab, migraine, pharmacokinetics, phase I, safety

## Abstract

Galcanezumab is used in several regions, including the United States, Europe, and China, as a preventive treatment for migraine. This study aimed to evaluate the safety, tolerability, and pharmacokinetics (PK) of galcanezumab in healthy Chinese participants. In this phase I single‐dose study, 30 healthy adults were assigned to one of the two cohorts (120 or 240 mg) and randomized in a 4:1 ratio to receive a single subcutaneous (SC) dose of galcanezumab or placebo. Overall, 29 (96.7%) participants reported 93 treatment‐emergent adverse events (TEAEs), with 21 participants reporting 44 TEAEs related to the study treatment. Most study‐related TEAEs (95%) were mild in severity. The most commonly reported TEAE was upper respiratory tract infection. The PK data demonstrated that maximum observed drug concentration (C_max_) and area under the serum concentration curve from time zero to infinity increased proportionally to dose, with an apparent clearance of 0.009 L/h and a terminal elimination half‐life (t_1/2_) of 27 days. Galcanezumab was safe and well tolerated and demonstrated a PK profile consistent with that of non‐Chinese populations, supporting its use for the preventive treatment of migraine in Chinese patients.

Migraine is a complex, multifactorial neurological disorder marked by recurrent, debilitating headaches, often accompanied by severe pain, nausea, vomiting, and sensitivity to light and sound, significantly affecting an individual's quality of life and daily activities.^1^, ^2^ The diverse and intricate nature of migraine's etiology, underlying mechanisms, and treatment approaches present substantial obstacles in migraine management.^1^ Findings from the Global Burden of Disease Study 2021 reported migraine as the second leading cause of disability‐adjusted life‐years among adults aged 20‐59 years globally (750.8 per 100,000 people),^3^ with a worldwide prevalence estimated to be 15.0%.^4^ The prevalence of migraine in China is reported to be up to 14.3%, similar to the global prevalence.^5^ In 2019, the prevalence of migraine in China reached 188.93 million, with a sharp rise in the number of cases between 2017 and 2019, indicating an increasing disease burden.^6^ This increase in prevalence is reflected in an analysis of Baidu Index data, exploring the spatiotemporal distribution of migraine in China.^6^

Galcanezumab is a humanized monoclonal antibody that selectively binds to calcitonin gene‐related peptide (CGRP), a neuropeptide involved in the pathophysiology of migraines.^7^, ^8^ Galcanezumab is used in several regions, including the United States, Europe, and China as a preventive treatment for migraine.^7^, ^9^ The European Headache Federation's 2022 consensus statement and the recent 2024 American Headache Society (AHS) position statement both recommend CGRP antagonists as the first‐line treatment for patients needing preventive migraine therapy without requiring two prior attempts with the standard‐of‐care treatment.^10^, ^11^

The phase III randomized PERSIST study demonstrated the sustained effectiveness and tolerability of galcanezumab in patients with episodic migraine from China, India, and Russia.^12^, ^13^ Phase II/III studies have also reported the efficacy, favorable safety, and tolerability of galcanezumab for up to 6 months to 1 year in patients with episodic and chronic migraines compared with placebo.^14^, ^15^, ^16^

A population pharmacokinetics (PK) analysis of seven phase I/III clinical trials was conducted, demonstrating that subcutaneous (SC) administration of galcanezumab could be described with a one‐compartment model with first‐order absorption and linear elimination.^17^ Galcanezumab's median time to maximum concentration (t_max_) was 5 days, the apparent clearance (CL/F) was 0.009 L/h, and the terminal elimination half‐life (t_1/2_) was 27 days. Patient body weight was determined not to have a clinically relevant impact on galcanezumab's PK; thus, dosing adjusted for body weight is not warranted in adults. Other covariates, including age, sex, immunogenicity, and injection‐site location, did not affect galcanezumab's PK. Additionally, over the dose range of 1‐600 mg, the maximum observed drug concentration (C_max_) and area under the serum concentration curve from time zero to infinity (AUC_0‐∞_) were generally proportional to dose in healthy non‐Chinese participants from the United States.^18^

The ethnic background of individuals might play a crucial role in understanding differences in drug safety, effectiveness, and dosage requirements across various regions.^19^ Here, we report the safety and PK properties of galcanezumab in a healthy Chinese population. The results from this study were used to support the dose selection of galcanezumab in Chinese patients for the preventive treatment of migraine in China.

## Methods

### Study Design

This was a phase I, single‐center, randomized, subject‐ and investigator‐blinded, placebo‐controlled, parallel‐group, single‐dose study conducted in healthy Chinese participants (NCT04085289). The study was conducted between November 2019 and May 2020 at Huashan Hospital, affiliated with Fudan University, Shanghai, China. All participants provided written informed consent before the study evaluations. The study was approved by the Institutional Review Board of Huashan Hospital, and conducted in accordance with the Declaration of Helsinki, the International Conference on Harmonization Good Clinical Practice guidelines, and local regulations.

### Participants

Healthy Chinese participants aged ≥18 years and a body mass index (BMI) ranging from 18.5 to 35.0 kg/m^2^ were included. Participants were required to be healthy, as confirmed by medical history, physical examination, vital signs, and clinical laboratory tests. Key exclusion criteria were any significant medical conditions, a history of substance abuse, and the use of medications that could interfere with the study outcomes.

### Randomization, Blinding, and Study Intervention

Participants were randomized in a 4:1 ratio to receive either galcanezumab or placebo as SC injections in the abdomen. Cohort A received a single dose of 120 mg galcanezumab (1 × 1 mL) or placebo (1 × 1 mL), while Cohort B received two injections of 120 mg galcanezumab or placebo (2 × 1 mL). The syringes containing either galcanezumab or placebo were visibly indistinguishable from each other. Each milliliter of galcanezumab solution contained 120 mg of galcanezumab; along with water and other excipients for injection.^20^

### Study Assessments

#### Bioanalytical Methods

The serum concentration of galcanezumab was analyzed using a validated enzyme‐linked immunosorbent assay (ELISA) method by WuXi AppTec (Shanghai, China). A MaxiSorp plate (Thermo Fisher Scientific, Waltham, MA) was coated with a solution of streptavidin and biotinylated CGRP. The plate was washed, and unadsorbed sites were blocked for 30 min using a blocking buffer at ambient room temperature on an orbital shaker. After blocking and washing the plate, analytes of standards, quality controls (QCs), and clinical samples were loaded onto the plate and incubated for 1 h. Following incubation, the plate was washed again, and horseradish peroxidase (HRP)‐conjugated mouse anti‐human immunoglobulin (Ig)G4 was added to the plate, which was incubated for 1 h. After the final wash, a signal was produced by adding tetramethylbenzidine peroxidase substrate. The absorbance was then measured at a wavelength of 450 nm with a background correction at 650 nm. The concentration of the samples was obtained from a standard curve with a weighting factor of 1/Y^2^; galcanezumab concentration is directly proportional to the absorbance measured in the wells of the plate. The lower and upper limits of quantitation were 0.75 and 30.00 ng/mL, respectively. The inter‐assay accuracy (relative error [%RE]) was −7.2% to 1.3%, and the inter‐assay precision (relative standard deviation [%RSD] or coefficient of variation [%CV]) was 3.8% to 7.5%. Serum samples above the upper limit of quantitation were determined by up to a 5000‐fold dilution with blank human serum.

#### Pharmacokinetic Methods and Analysis

Blood samples were collected at 8, 24, 48, 96, 120, 168, 216, 264, 336, 504, 672, 1008, 1344, 1680, 2016, 2688, and 3360 h postdose. The serum galcanezumab concentration–time data were used to calculate PK parameters including C_max_, AUC_0‐∞_, AUC from time zero to the last time point with a measurable concentration (AUC_0‐tlast_), percentage of AUC_0‐∞_extrapolated (%AUC_tlast‐∞_), t_max_, t_1/2_, CL/F, and apparent volume of distribution during the terminal phase (V_z_/F). The PK parameters were calculated using non‐compartmental analysis (Phoenix WinNonlin Version 8.1).

### Safety and Tolerability

Safety and tolerability were evaluated by monitoring adverse events (AEs), treatment‐emergent AEs (TEAEs), vital signs, clinical laboratory tests, and physical examinations. TEAEs were summarized based on treatment, severity, and their relationship to the study drug. The frequency of TEAEs, including the number of AEs, the number of participants experiencing an AE, and the percentage of participants experiencing an AE, was summarized by treatment, MedDRA Version 22.1 System Organ Class (SOC), and preferred term. Any serious adverse events (SAEs) were also tabulated.

### Immunogenicity

Blood samples were collected predose and on 15, 29, 57, 85, and 141 days postdose. An affinity capture elution (ACE) ELISA developed and validated by Lilly at Pacific Biomarkers Inc. (Seattle, WA) was used to screen and characterize anti‐drug antibodies (ADAs) to galcanezumab from the clinical samples. This ACE assay was based on the studies by Bourdage et al (2007)^21^ and Butterfield et al (2010),^22^ with an added acid pretreatment step. The frequency and percentage of participants with pre‐existing and treatment‐emergent ADAs (TE ADAs) to galcanezumab were summarized and listed for individual participants. TE ADA samples were defined as at least a four‐fold increase from baseline when ADAs were detected at baseline, referred to as treatment‐boosted ADA. If no ADAs were detected at baseline, treatment‐induced ADAs were defined as two‐fold (one dilution) greater than the minimum required dilution of the screening assay (1:10).

### Statistical Analysis

Descriptive statistics were used to summarize demographic characteristics, safety data, and PK parameters. Safety data were summarized using frequency counts and percentages.

## Results

Thirty healthy Chinese participants (76.7% male) with a mean age (standard deviation [SD]) of 27.6 ± 4.9 years, and a mean BMI of 24.1 ± 3.0 kg/m^2^ were randomized into the study and received at least one dose of galcanezumab or placebo (Table S1). Of these, 29 participants completed the study, and one participant discontinued due to refusal to attend the final follow‐up visit.

### Pharmacokinetics

**Figure 1:**
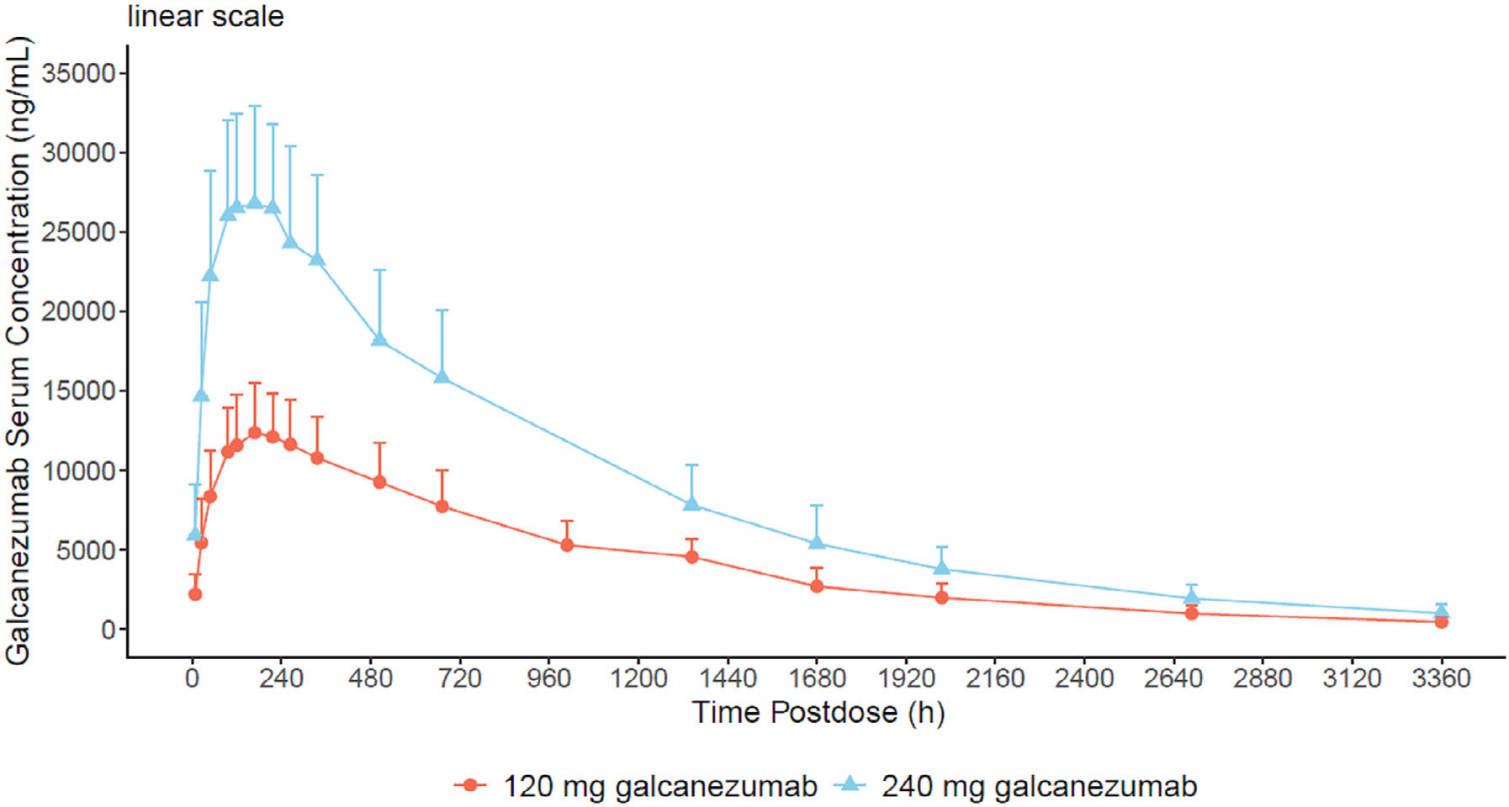
Pharmacokinetic profiles of galcanezumab in healthy Chinese participants. Data presented are arithmetic mean ± standard deviation.

The PK profiles of galcanezumab following a SC administration of 120 and 240 mg are illustrated in Figure 1, and the PK parameters are provided in Table 1.

**Table 1:** Pharmacokinetic Parameters of Galcanezumab

Pharmacokinetic Parameter	Galcanezumab 120 mg, N = 12	Galcanezumab 240 mg, N = 12^*^
AUC_(0‐tlast)_ (µg day/mL)^a^	566 (164)	1190 (306)
AUC_(0‐tlast)_ (µg day/mL)^b^	541 (34%)	1150 (26%)
AUC_(0‐∞)_ (µg day/mL)^a^	587 (177)	1230 (329)
AUC_(0‐∞)_ (µg day/mL)^b^	558 (36%)	1190 (27%)
C_max_ (µg/mL)^a^	12.9 (2.88)	27.8 (5.94)
C_max_ (µg/mL)^b^	12.6 (23%)	27.2 (23%)
t_max_ (day)^c^	7 (4‐14)	6 (2‐9)
t_1/2_ (day)^a^	26.9 (4.43)	28.0 (4.13)
t_1/2_ (day)^b^	26.6 (17%)	27.7 (15%)
CL/F (L/h)^a^	0.00951 (0.00377)	0.00865 (0.00224)
CL/F (L/h)^b^	0.00896 (36%)	0.00838 (27%)
V_z_/F (L)^a^	8.4 (1.8)	8.3 (1.99)
V_z_/F (L)^b^	8.2 (21%)	8.1 (23%)

AUC_(0‐∞)_, the area under the plasma concentration–time curves (AUC) from time zero extrapolated to infinity; AUC_(0‐tlast)_, area under the serum concentration curve (AUC) from zero to time corresponding to the last measurable concentration; CL/F, apparent total body clearance of drug calculated after extravenous administration; C_max_, maximum observed drug concentration; N, number of participants studied; t_1/2_, terminal half‐life; SD, standard deviation; t_max_, median time to maximum drug concentration; V_z_/F, apparent volume of distribution during the terminal phase after intravenous administration.

a Arithmetic mean (SD).

b Geometric mean (Geometric CV%).

c Median (minimum‐maximum).

* One subject from galcanezumab 240 mg treatment group withdrew with the last PK sample collected at 2688 h postdose (Day 113).

The C_max_ and AUC_0‐∞_ increased proportionally to dose, and the geometric mean CL/F and V_z_/F were approximately 0.009 L/h and 8 L, respectively. The median (minimum‐maximum) t_max_ was 7 (4‐14) days for galcanezumab at 120 mg and 6 (2‐9) days at 240 mg. Following t_max_, the serum concentrations of galcanezumab declined mono‐exponentially, with a mean t_1/2_ of about 26.6‐27.7 days (Table 1).

Thirty‐three planned PK sample collections were not obtained for analysis mostly due to COVID‐19. However, the absence of these samples did not significantly affect the PK interpretation, as the missing data occurred after t_max_, the AUC_tlast‐∞_ was minimal (<20%), and the t_1/2_ was estimated appropriately for all subjects.

### Safety and Tolerability

The safety analysis included all 30 participants. Overall, 29 participants (96.7%) reported 93 TEAEs, with 21 participants (70.0%) reporting 44 TEAEs related to the study drug (Table 2). Most study‐related TEAEs (95%) were mild in severity. Two TEAEs were moderate in severity: one case of upper respiratory tract infection (URTI) in the 120 mg group and one case of stye in the 240 mg groups. The incidence of TEAEs was lower in the placebo group (n = 2, 33.3%) compared to the 120 mg (n = 9, 75.0%) and 240 mg (n = 10, 83.3%) galcanezumab groups. No deaths or discontinuations were reported due to AEs during the study. Three participants receiving galcanezumab reported a SAE, two in the 120 mg group and one in the 240 mg group, but none were considered related to the study drug.

**Table 2:** Summary of Treatment‐Emergent Adverse Events in the Study Participants

	Galcanezumab (120 mg) (N = 12)	Galcanezumab (240 mg) (N = 12)	Placebo (N = 6)	Overall Population (N = 30)
**All AEs**				
Number of participants with AEs, n (%)	12 (100)	12 (100)	5 (83.3)	29 (96.7)
**Severity of AEs** ^*^				
Mild	35	39	13	87
Moderate	3	3	0	6
Severe	0	0	0	0
**Total**	38	42	13	93
**Number of participants with AEs related to study treatment (%)**	9 (75.0)	10 (83.3)	2 (33.3)	21 (70.0)
**Number of adverse events and severity** ^*^				
Mild	17	22	3	42
Moderate	1	1	0	2
Severe	0	0	0	0
**Total**	18	23	3	44

AEs, adverse events.

* Only the maximum severity of each adverse event is reported.

The most commonly reported TEAEs were infections and infestations, particularly URTI. The second most common TEAE was an increase in alanine aminotransferase (ALT) values (Table S2). No clinically significant safety‐related findings were observed in the clinical laboratory evaluations, vital sign measurements, electrocardiograms, or other observations. Three participants reported injection‐site reactions: one each in the placebo (mild erythema), 120 mg galcanezumab (mild erythema), and 240 mg galcanezumab (mild pain) groups. All TEAEs were mild and resolved without the need for concomitant treatment.

### Immunogenicity

Pre‐existing antibodies were found at baseline in 2 of the 6 participants (33.3%) in the placebo group and 2 of the 12 participants (16.7%) in the 120 mg group. No participant in the 240 mg group had pre‐existing antibodies at baseline. Of the 24 galcanezumab‐treated participants evaluated for TE ADAs, 5 were TE ADA‐positive post‐baseline: 3 (25.0%) in 120 mg group and 2 (16.7%) in the 240 mg group. The maximum ADA titers ranged from 1:20 to 1:640, with similar galcanezumab CL/F among participants with these titers (Table S3), demonstrating a lack of relationship between the immunogenicity titer and CL/F. Furthermore, the CL/F for all galcanezumab‐treated participants (Table 1) was similar to the CL/F for TE ADA‐positive participants (Table S3). All TE ADA‐positive participants were classified as treatment‐induced ADA. All participants in the placebo group were classified as TE ADA negative.

## Discussion

This phase I clinical study is the first to specifically evaluate the PK and safety of galcanezumab, administered as a single SC dose of 120 or 240 mg in healthy Chinese participants. The results demonstrated that the PK of galcanezumab in Chinese participants were comparable to that in non‐Chinese participants. The PK results of this study were consistent with a population PK model analysis based on seven phase I/III clinical trials of galcanezumab, conducted primarily in a global non‐Chinese population that included healthy volunteers and migraine patients.^17^ Our findings support the view that therapeutic monoclonal antibodies are less likely to be influenced by intrinsic or extrinsic ethnic factors compared to small molecular compounds.^23^, ^24^ Therefore, galcanezumab can be administered without the need for dose adjustments based on ethnicity.

The safety and tolerability profile of galcanezumab was favorable; there were no deaths or discontinuations due to AEs reported, and most TEAEs were mild in severity. Previous safety and tolerability findings in patients with episodic or chronic migraine showed that galcanezumab was well tolerated in real‐life settings, with a safety profile consistent with that observed in randomized controlled trials.^8^, ^25^ In the phase III randomized PERSIST study, galcanezumab demonstrated sustained effectiveness and tolerability in patients with episodic migraine from China, India, and Russia.^12^, ^13^ The safety findings from our study aligned with the established long‐term safety profile of galcanezumab, with no patients reporting any SAEs related to the treatment.^12^, ^13^ Common TEAEs observed in PERSIST study were injection‐site reactions, injection‐site pruritus, and URTI.^12^, ^13^ Increased ALT and URTI were the most common TEAEs reported in our study.

Immunogenicity results in Chinese participants did not appear to influence the PK of galcanezumab, which is consistent with previous findings in non‐Chinese participants.^17^ A small proportion of participants developed TE ADAs, but the impact of immunogenicity on the PK of galcanezumab was not observed. Overall, the safety, tolerability, and PK observed in healthy Chinese participants support the use of galcanezumab for the preventive treatment of migraine in China, potentially offering a new therapeutic option for patients with migraine.

The strengths of this study include its rigorous design (randomization, blinding, and the use of a placebo control group) and the comprehensive assessment of safety and PK conducted, which provided a thorough evaluation of galcanezumab in the target population. However, the study's limitations include its short follow‐up duration and single‐center design, which may limit the generalizability of the findings.

## Conclusion

Galcanezumab was safe and well tolerated, demonstrating a PK profile consistent with observed in non‐Chinese populations. The results of this study support the use of galcanezumab for the preventive treatment of migraine in Chinese patients following its recent regulatory approval in China.

## Conflicts of Interest

William Kielbasa, Chenxi Qian, and Yanjie Zhang are employees of Eli Lilly and Company. The other authors have no conflicts of interest to disclose.

## Funding

Funding was provided by Eli Lilly and Company.

## Supporting information



Supporting Information
