# Supplementary material for: Safety, Tolerability, and Pharmacokinetics of Galcanezumab, an Anti‐CGRP Antibody, in Healthy Chinese Participants
Source: Clin Pharmacol Drug Dev. 2025 Sep 12;14(12):918–24. doi: 10.1002/cpdd.1599 (PMC12672407; doi:10.1002/cpdd.1599)
Supplement: Supplementary file 1 — Supporting Information [file CPDD-14-918-s001.docx]

**Supplementary Table 1. Demographics of the study participants**

|  | **Galcanezumab**  **(120 mg)**  **(N=12)** | **Galcanezumab**  **(240 mg)**  **(N=12)** | **Placebo**  **(N=6)** | **Overall population**  **(N=30)** |
| --- | --- | --- | --- | --- |
| **Age, years, mean (SD)** | 26.8 (6.0) | 28.8 (4.1) | 26.7 (4.2) | 27.6 (4.9) |
| **Sex, n (%)** | | | | |
| Male | 8 (66.7%) | 11 (91.7%) | 4 (66.7%) | 23 (76.7%) |
| Female | 4 (33.3%) | 1 (8.3%) | 2 (33.3%) | 7 (23.3%) |
| **Ethnicity – Not Hispanic or Latino (%)** | 100 | 100 | 100 | 100 |
| **Race – Asian Chinese (%)** | 100 | 100 | 100 | 100 |
| **Body weight, kg, mean (SD)** | 66.0 (9.4) | 69.0 (9.1) | 63.7 (14.4) | 66.7 (10.3) |
| **Height, cm, mean (SD)** | 166.0 (7.5) | 167.2 (6.3) | 165.4(6.4) | 166.4 (6.6) |
| **Body mass index, kg/m^2^, mean (SD)** | 23.9 (3.2) | 24.6 (2.2) | 23.1 (4.2) | 24.1 (3.0) |

Abbreviations: N, number of participants studied; SD, standard deviation.

**Supplementary Table 2. Frequency of treatment-emergent adverse events related to study treatment in the study participants**

|  | **Number of adverse events^*^; number of participants with adverse events (participants with adverse events, %)** | | | |
| --- | --- | --- | --- | --- |
| **Treatment-emergent adverse events** | **Galcanezumab**  **(120 mg)**  **(N=12)** | **Galcanezumab**  **(240 mg)**  **(N=12)** | **Placebo**  **(N=6)** | **Overall population**  **(N=30)** |
| **Infections and infestations** | | | | |
| Upper respiratory  tract infection | 6; 4 (33.3) | 2; 2 (16.7) | - | 8; 6 (20.0) |
| Stye | - | 1; 1 (8.3) | - | 1; 1 (3.3) |
| **Total** | 6; 4 (33.3) | 3; 3 (25.0) | - | 9; 7 (23.3) |
| **Laboratory findings** |  |  |  |  |
| Alanine  aminotransferase  increased | - | 4; 4 (33.3) | - | 4; 4 (13.3) |
| Blood pressure increased | - | 3; 1 (8.3) | - | 3; 1 (3.3) |
| Aspartate aminotransferase increased | - | 2; 2 (16.7) | - | 2; 2 (6.7) |
| **Total** | - | 9; 5 (41.7) | - | 9; 5 (16.7) |
| **Gastrointestinal disorders** | | | | |
| Aphthous ulcer | 1; 1 (8.3) | 3; 1 (8.3) | - | 4; 2 (6.7) |
| Dry mouth | 1; 1 (8.3) | - | 1; 1 (16.7%) | 2; 2 (6.7) |
| Abdominal pain | 1; 1 (8.3) | - | - | 1; 1 (3.3) |
| Constipation | 1; 1 (8.3) | - | - | 1; 1 (3.3) |
| **Total** | 4; 4 (33.3) | 3; 1 (8.3) | 1; 1(16.7) | 8; 6 (20.0) |
| **Respiratory, thoracic and mediastinal disorders** | | | | |
| Nasal obstruction | 1; 1 (8.3) | 2; 2 (16.7) | - | 3; 3 (10.0) |
| Oropharyngeal pain | 2; 2 (16.7) | - | 1; 1 (16.7) | 3; 3 (10.0) |
| Oropharyngeal discomfort | 1; 1 (8.3) | - | - | 1; 1 (3.3) |
| **Total** | 4; 4 (33.3) | 2; 2 (16.7) | 1; 1 (16.7) | 7; 7 (23.3) |
| **General disorders and administration site conditions** | | | | |
| Injection site reaction | 1; 1 (8.3) | 2; 1 (8.3) | 1; 1 (16.7) | 4; 3 (10.0) |
| Asthenia | - | 1; 1 (8.3) | - | 1; 1 (3.3) |
| Injection site  hemorrhage | - | 1; 1 (8.3) | - | 1; 1 (3.3) |
| **Total** | 1; 1 (8.3) | 4; 2 (16.7) | 1; 1 (16.7) | 6; 4 (13.3) |
| **Nervous system disorders** | | | | |
| Headache | 1; 1 (8.3) | - | - | 1; 1 (3.3) |
| Hypoesthesia | - | 1; 1 (8.3) | - | 1; 1 (3.3) |
| **Total** | 1; 1 (8.3) | 1; 1 (8.3) | - | 2; 2 (6.7) |
| **Eye disorders** | | | | |
| Dry eye | 1; 1 (8.3) | - | - | 1; 1 (3.3) |
| Eye pain | 1; 1 (8.3) | - | - | 1; 1 (3.3) |
| **Total** | 2; 1 (8.3) | - | - | 2; 1 (3.3) |
| **Musculoskeletal and connective tissue disorders** | | | | |
| Pain in extremity | - | 1; 1 (8.3) | - | 1; 1 (3.3) |
| **Total** | - | 1; 1 (8.3) | - | 1; 1 (3.3) |
| **Overall Total** | 18; 9 (75.0) | 23; 10 (83.3) | 3; 2 (33.3) | 44; 21 (70.0) |

* Adverse events with a change in severity are only counted once at the highest severity MedDRA version 22.1.

Abbreviations: N, number of participants studied.

**Supplementary Table 3. Clearance of galcanezumab in participants classified as treatment-emergent anti-drug antibody**-**positive post-baseline**

| **Galcanezumab treatment group** | **Galcanezumab CL/F (L/h)** | **Maximum ADA Titer** |
| --- | --- | --- |
| 120 mg | 0.0120 | 1:20 |
| 120 mg | 0.0102 | 1:20 |
| 120 mg | 0.00900 | 1:20 |
| 240 mg | 0.0130 | 1:40 |
| 240 mg | 0.00874 | 1:640 |

Abbreviations: ADA, anti-drug antibody; CL/F, apparent total body clearance; TE ADA, treatment-emergent anti-drug antibody.

**Author Contributions**

All authors made substantial contributions to conception and design, acquisition of data, or analysis and interpretation of data; took part in drafting the article or revising it critically for important intellectual content; and agreed to be accountable for all aspects of the work. William Kielbasa, Chenxi Qian, Yanjie Zhang and Liang Wang contributed to conception of the work, design and supervision. Jingjing Wang, Nanyang Li, Jinjie He, Jing Zhang, Lili Wang, Chenxi Qian, and Yanjie Zhang were involved in data acquisition and interpretation. Chenxi Qian was responsible for data analysis. All the authors revised the manuscript critically and approved the final version of the manuscript.

**Data Availability**

Lilly provides access to all individual participant data collected during the trial, after anonymization, with the exception of pharmacokinetic or genetic data. Data are available upon reasonable request. Access is provided after a proposal has been approved by an independent review committee identified for this purpose and after receipt of a signed data sharing agreement. Data and documents, including the study protocol, statistical analysis plan, clinical study report, blank or annotated case report forms, will be provided in a secure data sharing environment. For details on submitting a request, see the instructions provided at [www.vivli.org](http://www.vivli.org).
